# Mechanistic Insights Into Supercapacitive Swing Adsorption via Acid–Base Titrations

**DOI:** 10.1002/smll.73437

**Published:** 2026-04-20

**Authors:** Fareed Ul‐Haq Khan, Kai Landskron

**Affiliations:** ^1^ Department of Chemistry Lehigh University Bethlehem Pennsylvania USA

**Keywords:** carbon capture, electrochemistry, supercapacitive swing adsorption, titrations

## Abstract

The mechanism of supercapacitive swing adsorption (SSA) has been investigated by pH measurements and acid‐base titrations of water‐extracted, charged activated carbon electrodes at voltage windows varying from 0–0.5 to 0–1.4 V. 15%CO_2_/85%N_2_ mixtures and pure N_2_ were used as feed gases. Deionized water extracts from negatively charged electrodes showed a pH increase for both feed gases. Titration of extracts from negative electrodes exposed to CO_2_ consumed more HCl titrant compared to those without CO_2_. Conversely, extracts from the positive electrodes consumed more KOH titrant when only N_2_ was present. The pH values and titrant amounts consumed increased with increasing voltage window for both electrodes. Larger amounts of acid consumed for negative electrode extracts in the experiments with CO_2_ are explained by the adsorption of additional protons created from CO_2_ hydrolysis. Smaller amounts of base are consumed for the positive electrode in the presence of CO_2_ due to the reaction of bicarbonate with acid generated at the positive electrode. The results explain that CO_2_ is adsorbed to the negative electrode and desorbed from the positive electrode upon charging. They are evidence for the ionic liquid–solid mechanism of SSA, which assumes that CO_2_ sorption is driven by selective proton adsorption to the negative electrode.

## Graphical abstract

The mechanism of supercapacitive swing adsorption (SSA) for carbon dioxide (CO_2_) capture has been determined using pH titration of charged activated carbon electrode extracts under different voltage windows. Clear pH differences were observed at the negative (gas exposed) and positive electrode extracts, indicating protonation at the negative electrode and migration of bicarbonates/carbonates towards the positive electrode.

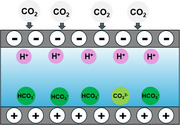

## 1 Introduction

Rising atmospheric carbon dioxide levels since the industrial revolution have driven intense research in carbon capture technologies to mitigate climate change [1]. To limit the increase of the global temperature to 1.5°C, achieving net‐zero CO_2_ emissions by 2050 is considered critical [2]. Carbon capture from point sources and direct air capture (DAC) are among the proposed solutions [3, 4]. An indirect air capture method is ocean capture, where CO_2_ is extracted from seawater, thereby enhancing the ocean's capacity to absorb additional CO_2_ from the atmosphere to maintain equilibrium between atmospheric and oceanic CO_2_ levels [5]. Amine‐scrubbing is the most established CO_2_ separation technology, owing to its high CO_2_ absorption capacity and selectivity, but it suffers from the volatility, toxicity, and corrosiveness of amines, and the high regeneration energy requirements [6, 7, 8]. These issues have spurred the development of alternative CO_2_ capture methods, including temperature‐swing adsorption [9] and pressure‐swing adsorption [10] using solid sorbents. Membrane‐based separations have also been explored [11].

**SCHEME 1:**
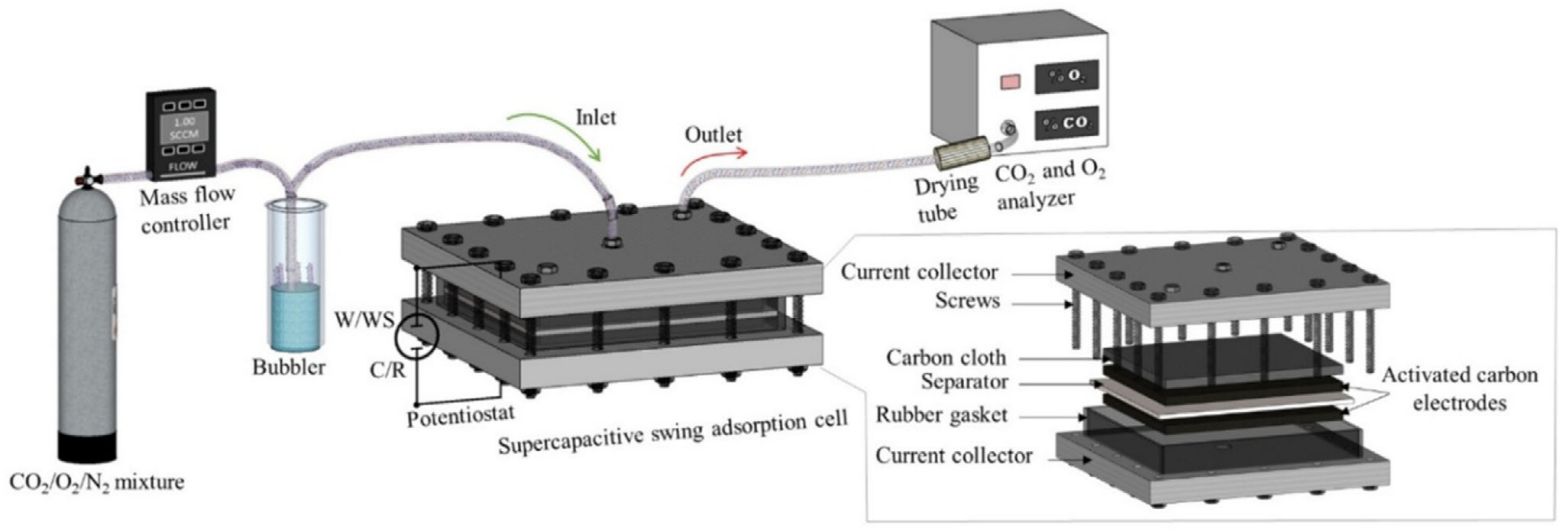
Supercapacitive swing adsorption experimental setup showing the SSA cell and its components. Reproduced with permission [21].

Electrochemical CO_2_ capture has emerged as a promising alternative that does not require temperature and pressure changes or differentials. Among electrochemical approaches, supercapacitive swing adsorption (SSA) is a recently developed method that uses the charge and discharge of supercapacitor electrodes to capture and release CO_2_ [12]. In SSA, two activated carbon electrodes separated by a filter paper infiltrated by an aqueous electrolyte such as NaCl or MgCl_2_ are charged with a small voltage in the order of 1 V [13], Scheme 1. A carbon cloth is interfaced as a gas diffusion layer between one of the electrodes and one of the current collector end plates. During charging, CO_2_ from a gas mixture is flown radially through the gas diffusion layer. CO_2_ is selectively adsorbed to the gas‐exposed electrode in the cell, when this electrode is the negative electrode; upon discharge, the CO_2_ is released. It has been further observed that when the gas‐exposed electrode is the positive electrode, CO_2_ is desorbed upon charging, and is adsorbed during discharging [14, 15, 16, 17]. Charging and discharging between −1 and +1 V instead of −1 and 0 V increases the amounts that can be reversibly adsorbed, but at the expense of a higher energy consumption [18]. While significant progress has been made to improve the adsorption capacity, energy consumption, and charge‐discharge protocols [19, 20, 21, 22], there is currently no detailed understanding of the mechanism of SSA [23, 24].

**FIGURE 1:**
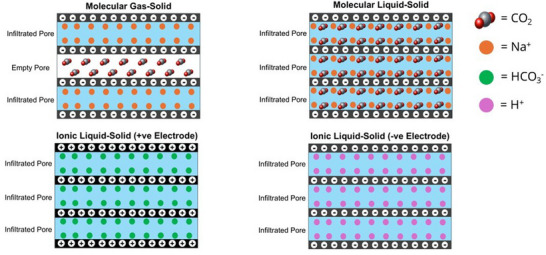
Schematic illustration of proposed mechanisms for supercapacitive swing adsorption (SSA) of CO_2_ at activated carbon electrodes. The schematics illustrate possible charge‐dependent interactions between CO_2_, the electrolyte, and the electrode surfaces during charging and discharging.

Several mechanisms have been proposed to explain the SSA phenomenon [14, 16], Figure 1. The initially proposed one was the molecular gas–solid mechanism, where neutral CO_2_ molecules are physically adsorbed within hydrophobic pores that are not filled with electrolyte. The adsorptivity is different between the charged and the uncharged state due to the different Fermi levels in the delocalized π‐electron system. Because CO_2_ has Lewis acidic properties, it interacts more strongly with the more Lewis‐basic, electron‐rich, negatively charged carbon π‐system, explaining CO_2_ adsorption to the negative electrode. Vice versa, the positive, electron‐poorer carbon electrode is less Lewis‐basic than a neutral electrode, hence it desorbs CO_2_ when charged. However, physisorption of CO_2_ at a gas‐solid interface is known to be fast, which is inconsistent with the observed relatively long cycle times, which vary between 1–5 h depending on the gas flow rate and the CO_2_ concentration in the feed gas [25]. This pointed toward mechanisms at the liquid‐solid interface that involve CO_2_ dissolution and diffusion through the liquid electrolyte. In the proposed molecular liquid–solid mechanism, dissolved molecular CO_2_ is adsorbed to the electric double layer due to differences in solubility between the double layer and the bulk electrolyte, and/or electric field gradients within the double layer, leading to enhanced accumulation at charged interfaces. Augustyn and co‐workers recently found support for this mechanism, but used gold electrodes, which are chemically very different from activated carbon electrodes [26]. A third proposed mechanism is the ionic liquid–solid mechanism, in which CO_2_ dissolves and hydrolyzes to form H^+^, HCO_3,_^−^ and CO_3_^2−^ ions (see Equations (1), (2), (3), (4)).

(1) $$C{O_{2}}_{\left(g\right)}⇄C{O_{2}}_{\left(aq\right)}$$

(2) $$C{O_{2}}_{\left(aq\right)}+H_{2}O_{\left(l\right)}⇄H_{2}C{O_{3}}_{\left(aq\right)}$$

(3) $$H_{2}C{O_{3}}_{\left(aq\right)}⇄HCO_{3\left(aq\right)}^{-}+{H^{+}}_{\left(aq\right)}$$

(4) $$HCO_{3\left(aq\right)}^{-}⇄CO_{3\left(aq\right)}^{2-}+{H^{+}}_{\left(aq\right)}$$

**FIGURE 2:**
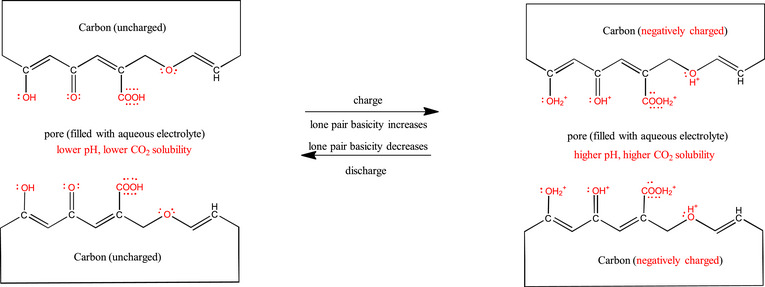
Ionic liquid–solid mechanism at the negative electrode driven by direct protonation of electron lone pairs at O heteroatoms of functional groups on the pore surfaces. The basicity of the heteroatoms increases upon charging due to electron‐electron repulsion.

These ions are then adsorbed at the charged electrodes: protons at the negatively charged electrode, and bicarbonate/carbonate at the positively charged one, Figure 1. Protons may be adsorbed via direct protonation of electron lone pairs at the surface functional groups, for example, keto, hydroxo, or carboxylic acid groups [27]. The electron lone pairs are assumed to increase in their basicity upon negative charging due to enhanced electron‐electron repulsion (Figure 2). In addition to oxygen‐containing functional groups, the polarized π‐electron system of the carbon framework may contribute to the enhanced proton affinity under negative charging. The enhanced proton adsorption increases the pH near the negative electrode, leading to enhanced CO_2_ solubility near the negative electrode, and more CO_2_ is absorbed from the gas phase into the electrolyte near the negative electrode. Forse and co‐workers recently postulated, electrostatic repulsion of anionic HCO_3_^−^ and CO_3_^2−^ ions from the negative electrode may lead to CO_2_ depletion at the negative electrode, driving further gas dissolution from the bulk phase [28]. These mechanisms are not mutually exclusive and may operate concurrently depending on electrode materials, electrolyte composition, and charging conditions.

If the ionic liquid–solid mechanism was correct, then the electrolyte within the electrode's pores should undergo a pH change upon charging. Assuming a pH‐neutral electrolyte, the adsorption of H^+^ to the negative electrode would argue for an increase of pH in the bulk electrolyte outside the double layer (Figure 2). The adsorption of bicarbonate/carbonate to the double‐layer of the positive electrode would be expected to decrease the pH, because base is adsorbed to the double layer, and the protons from CO_2_ hydrolysis are left in the bulk solution outside the double‐layer. At the same time, OH‐groups on the activated carbon surface may increase in acidity due to repulsion between positive charges.

To test the pH change hypothesis, we charged an SSA module with a radial gas flow system (Scheme 1) in a 15% CO_2_/85% N_2_ gas stream and then disassembled the module in the charged state. We immediately isolated the electrodes and extracted the electrolyte within the electrode with deionized water. We then measured the pH of the extract and subsequently titrated it to determine the amount of acid/base in the electrode extract. As a control, we also did analogous experiments in a pure N_2_ atmosphere. Experiments at different voltage windows were also done to investigate the effect of voltage on pH. Previous work has shown that there is a positive correlation between the voltage window and sorption capacity [20]. Finally, we also investigated the electrode extracts at the end of the discharge half‐cycles. Experimental methods are available in the supporting information.

## 2 Results and Discussion

### 2.1 pH Changes at the Electrodes After Charging

The extracts from the charged electrodes showed noticeably different pH values depending on whether CO_2_ had been present during charging, and whether the electrode was positively or negatively charged (see supporting information for sample preparation). The “never‐charged” reference electrode produced a mildly acidic extract with a pH of 5.3, likely due to acidic surface functionalities such as carboxylic acid and phenolic groups releasing some H^+^. The negative electrode exhibited a large pH increase in both N_2_ and CO_2_/N_2_ atmospheres, whereby the increase was greater in N_2_ (pH 9.7) compared to CO_2_ (pH 9.1). The increase of the pH in the presence of N_2_ is explained by adsorption of H^+^ to negatively charged carbon (C^x−^) due to water autoprotolysis, leading to the deprotonation of water molecules to form hydroxide ions, and subsequent reestablishment of the chemical equilibrium Equations (5),(6). The dissociation of the water molecules into H^+^ and OH^−^ may be promoted by the strong electric field gradient in the double layer, according to the Wien effect [29].

(5) $$H_{2}O_{\left(l\right)}\to H_{\left(aq\right)}^{+}+OH_{\left(aq\right)}^{-}$$

(6) $$H_{\left(aq\right)}^{+}+Carbon_{\left(s\right)}^{x-}\to CarbonH_{\left(s\right)}^{\left(x-1\right)-}$$

In addition, the acidity of acidic pore surface functionalities decreases because of the electrostatic attraction between the acidic protons and negative charges in the electrodes. The somewhat lower pH in the presence of CO_2_ is consistent with the acidic properties of hydrated CO_2_, which provides additional protons that can adsorb to the negative electrode (Equation 7).

(7) $$H_{2}C{O_{3}}_{\left(aq\right)}^{*}+Carbon_{\left(s\right)}^{x-}\to CarbonH_{\left(s\right)}^{\left(x-1\right)-}+HC{O_{3}}_{\left(aq\right)}^{-}$$

The produced weakly basic bicarbonate and carbonate ions located in the bulk electrolyte have a buffering effect on the pH, thereby somewhat lowering the pH Equation (8).

(8) $$HC{O_{3}}_{\left(aq\right)}^{-}+OH_{\left(aq\right)}^{-}\to C{O_{3}}_{\left(aq\right)}^{2-}$$

Because the applied potential largely drops across the electrical double layers rather than across the separator, there is only a limited electrostatic driving force for long‐range migration of bicarbonate/carbonate ions through the bulk electrolyte. It is noteworthy that the pH increase occurs despite the presence of the very concentrated 3 M MgCl_2_ electrolyte. This argues that the electrode has very high selectivity for proton adsorption over Mg^2+^ adsorption. This high preference is explained by the direct protonation of Bronsted‐basic sites at the negative electrodes, such as electron lone pairs of oxo‐ and hydroxo groups that increase in energy and basicity upon charging due to increased electron‐electron repulsion, Figure 2. Direct protonation is energetically favored over Mg^2+^ adsorption because charge separation is minimized. Mg^2+^ cannot easily bind directly via coordinative, covalent bonds because of the required energetically unfavorable desolvation of the Mg(H_2_O)_6_^2+^ ions Equation (9) [30].

(9) $$Mg{\left(H_{2}O\right)}_{6\left(aq\right)}^{2+}+Carbon_{\left(s\right)}^{x-}\to {\left(H_{2}O\right)}_{5}MgCarbon_{\left(s\right)}^{\left(x-2\right)-}+H_{2}O_{\left(aq\right)}$$

Steric hindrance effects inside small micropores of the activated carbon may further contribute to the proton selectivity [31]. The direct protonation mechanism explains our previous observation that the CO_2_ adsorptivity is largely independent from the metal cations in the electrolyte and does not decrease upon increase of the electrolyte concentration due to ion competition [16]. Like Mg^2+^, other cations like Li^+^, Na^+^, and K^+^ would need unfavorable desolvation for direct coordination to the negative electrode, hence protons are preferred over these ions.

For the positive electrode exposed to N_2_ only, the extract was highly acidic with an initial pH of ∼2.1. Similarly, the extract from the positive electrode charged in the presence of CO_2_ showed a pH of ∼2.5. Both are significantly more acidic than the never‐charged electrode (pH ∼5.3). For experiments in N_2_, the acidity can be explained by the increased acidity of acidic protons on the pore surfaces due to electrostatic repulsion between the acidic protons and the positive charges in the carbon. Further, some OH^−^ from the water electric field promoted autoprotolysis in the double‐layer may adsorb to the electrical double layer of the positive electrode, leading to the release of protons from water molecules in the bulk solution to reestablish the chemical equilibrium Equation (10).

(10) $$OH_{\left(aq\right)}^{-}+Carbon_{\left(s\right)}^{x+}\to {\left(OH^{-}\right)}_{ads}Carbon_{\left(s\right)}^{x+}$$

In the presence of CO_2_, the pH is slightly higher possibly due to migration of some bicarbonate ions from the negative electrode to the positive electrode, where some of the protons generated at the positive electrode react with the HCO_3_^−^ to form CO_2_ gas and H_2_O. (Equation 11). The generated gaseous CO_2_ diminishes the overall sorption capacity. The idea that the positive electrode partly diminishes the adsorptive effects at the negative electrode is further supported by a recent study by Forse et al. who replaced the positive activated carbon electrode by a zinc foil, and observed an increased overall sorption capacity [32].

(11) $$HC{O_{3}}_{\left(aq\right)}^{-}+H_{\left(aq\right)}^{+}\to H_{2}O_{\left(l\right)}+C{O_{2}}_{\left(aq\right)}$$

The adsorption of significant amounts of bicarbonate or carbonate to the positive electrode is not supported because this would lead to a depletion of these basic species in the bulk solution, and a lower pH compared to N_2_. However, we observe a slightly higher pH in a CO_2_ environment compared to N_2_, meaning the bicarbonate ions do not preferentially bind at the positive electrode and are outnumbered by Cl^−^ ions. This argues that bicarbonate and carbonate adsorption to the double‐layer of the positive electrode is not significant to the SSA effect, and the double layer at the positive electrode is mainly made of Cl^−^ ions.

### 2.2 Titration Measurements of Adsorbed Species

Titration of the electrode extracts provides a quantitative measure of the amount of acid and base produced at the electrodes upon charging. The amount of titrant consumed (TC) can be calculated using Equation (12), where V_T_ (mL) is the volume of the acid or base needed to neutralize the electrode solution, C_T_ (mM) is the concentration of the titrant used, and m (kg) is the mass of the electrode.

(12) $$TC=\frac{V_{T}\times C_{T}}{m}$$

**FIGURE 3:**
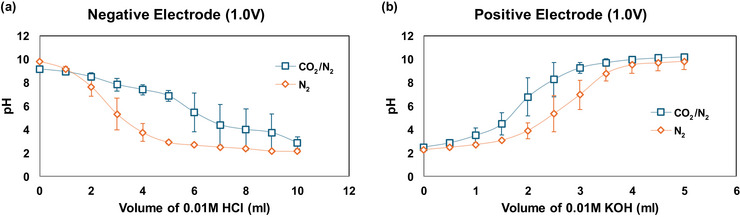
pH titration curves at 1.0 V voltage window for (a) negative and (b) positive electrodes showing the titration curves in the case of CO_2_/N_2_ mixture (blue) and pure N_2_ (orange). The error bars represent the average and uncertainty from three trials.

For the negative electrode at 1 V, the amount of acid required to reach the equivalence point was 203 mmol of HCl per kg of electrode in the presence of N_2_ only, versus 452 mmol/kg in the presence of CO_2_ (Figure 3). This means that the CO_2_‐exposed negative electrode stored a larger amount of base than the negative electrode charged without CO_2_. The titration curve for CO_2_ showed a region where the pH remains relatively constant despite the addition of acid. This behavior is characteristic of a bicarbonate/carbonate buffer present in the solution.

The inflection (= equivalence) point (occurring at a pH of about 6) in the acidic region indicates the presence of a weak base, further corroborating the presence of (bi)‐carbonate. In contrast, for N_2_, no buffer zone was observed, and the inflection point was near neutral pH due to the absence of the weakly basic HCO_3_^−^ and CO_3_^2−^ions. The much higher acid consumption for the negative electrode in the presence of CO_2_ is explained by the adsorption of protons generated from the CO_2_ hydration to the negative electrode, leaving bicarbonate and carbonate species in the bulk electrolyte within the electrode.

The positive electrode showed a different trend. The extract from the positive electrode charged in N_2_ required 282 mmol of KOH per kg of electrode to reach the equivalence point, whereas the positive electrode charged in CO_2_ required roughly 179 mmol/kg of KOH. In this case, there is no pronounced buffering effect indicating that no significant amounts of (bi)‐carbonate species are present. This is plausible because the positive electrode is not gas‐exposed, hindering CO_2_ dissolution and hydration. Any (bi)‐carbonate migrated from the negative to the positive electrode is likely converted to gaseous CO_2_ during the charging step as explained above (Equation 11). Overall, the results from the titrations further confirm that the electrical double‐layer at the positive electrode unlikely contains appreciable amounts of (bi)‐carbonate, but Cl^−^, and that CO_2_ sorption takes place predominantly near the negative electrode via the direct protonation mechanism.

As a reference, an electrolyte‐infiltrated electrode that was never charged was also extracted. The electrode extract (having a pH of 5.3) required very little titrant (less than 10 mmol/kg of KOH), showing that the previously observed large pH changes are due to the charging and not inherent due to the carbon's intrinsic functional groups.

**FIGURE 4:**
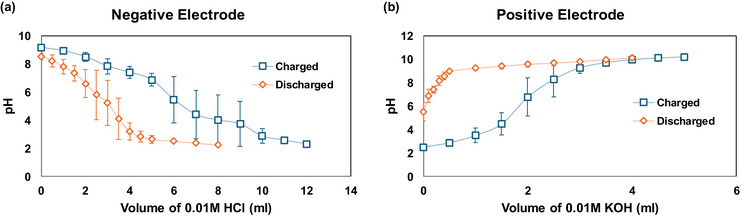
Titration curves for (a) negative and (b) positive electrode extracts after charging (half‐cycle, blue) and discharging (full cycle, orange). The error bars represent the average and uncertainty from three trials.

Next, discharged electrodes were extracted after ten full charge‐discharge cycles, and the pH was measured. The extract from the negative electrode showed a pH of 8.5 after discharge. This is smaller than the pH of 9.2 measured for the extract from the charged electrode, but larger than the pH of the electrode extract of the electrode that was never charged (pH 5.3). The extract from the positive electrode was at pH 5.5 after discharge which is higher than the extract of the charged electrode (pH 2.5) (Figure 4). This is very close to the pH of the extract from the electrode, which was never charged.

The titrant consumption for the discharged negative electrode extract was 262 mmol/kg compared to 452 mmol/kg for the charged one, and the difference (190 mmol/kg) can be attributed to the cycled (bi)‐carbonate. This value is significantly larger than the CO_2_ adsorption capacity measured with the CO_2_ analyzer (82 mmol/kg). This can be explained by the effect at the positive electrode. At the discharged positive electrode, the pH is near neutral and very little base is consumed (10 mmol). The difference of the titrant amounts in the charged versus the discharged state is (179–10) mmol/kg = 169 mmol/kg, which may be attributed to the CO_2_ amounts desorbed at the positive electrode during charging. This is nearly as much as the 190 mmol/kg adsorbed at the negative electrode, arguing that adsorption and desorption nearly cancel out. However, because only the negative electrode is gas‐exposed, the adsorption at the negative electrode is faster than the desorption at the positive electrode, and hence a significant adsorptive effect is still present (82 mmol/kg).

### 2.3 Effects of Voltage Window

**FIGURE 5:**
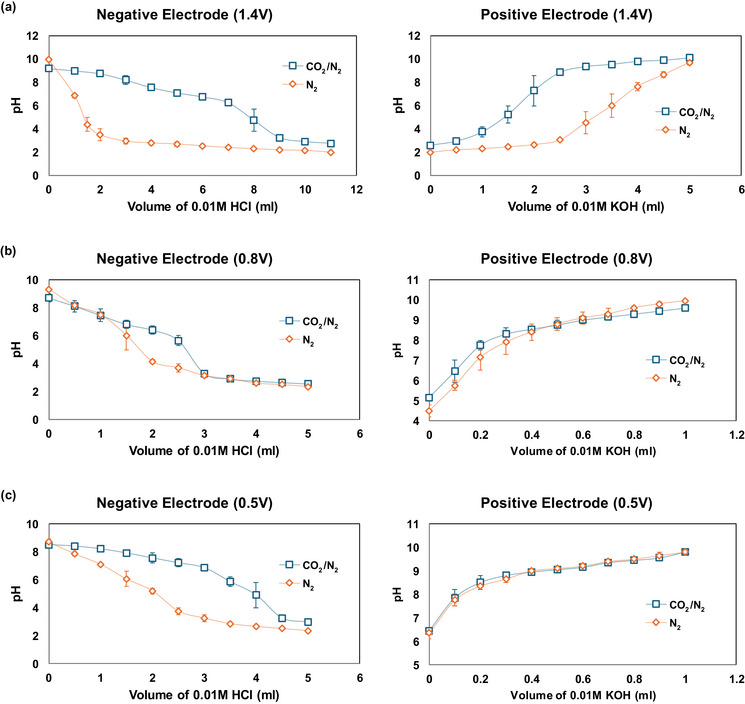
pH titration curves for (a) 1.4 V, (b) 0.8 V, and (c) 0.5 V, showing the effect of voltage window on pH and titrant consumption for CO_2_/N_2_ mixture (blue) and pure N_2_ (orange). The error bars represent the average and uncertainty from three trials.

In a previous study, we observed that the CO_2_ sorption capacity increases with the voltage window [20]. This prompted us to investigate if the pH changes and the titrant consumed follow this trend when the voltage was increased from −0.5 to −1.4 V. In all experiments, the capacitive behavior of the system was verified (Figures S3 and S4), and the increase in the sorption capacity with the voltage was confirmed (Table S1). The pH of the negative electrode extract after charging was basic at all investigated voltages and increased with increasing voltage (Table 1). The titrant consumption declined from 1.4 to 0.5 V and was 578 mmol/kg at 1.4 V, 306 mmol/kg at 0.8 V, and 189 mmol/kg at 0.5 V (Figure 5), which is in accordance with decreasing sorption capacity. A comparison of the titrant consumed to reach the equilibrium point can be seen in Table 2. The higher titrant consumption at 1.4 V may also have a contribution from beginning water electrolysis, producing OH^−^ at the negative electrode. This interpretation is supported by an increase in current in the cyclic voltammetry curves (Figure S3).

**TABLE 1:** pH values of electrode extracts after charging at different voltages.

Voltage window	Negative electrode		Positive electrode	
	Pure N_2_	CO_2_/N_2_	Pure N_2_	CO_2_/N_2_
Not charged	5.30 ± 0.30			
0.5V	8.70 ± 0.20	8.50 ± 0.15	6.30 ± 0.25	6.45 ± 0.10
0.8V	9.30 ± 0.10	8.70 ± 0.30	4.50 ± 0.30	5.15 ± 0.20
1.0V	9.75 ± 0.15	9.10 ± 0.25	2.10 ± 0.20	2.50 ± 0.15
1.4V	9.95 ± 0.10	9.20 ± 0.20	2.00 ± 0.10	2.60 ± 0.10
1.0 V (discharged)		8.53 ± 0.25		5.50 ± 0.78

**TABLE 2:** Amount of titrant consumed during acid‐base titration of electrode extracts at different voltage windows (0.01 M HCl used to titrate negative electrode extract, 0.01 M KOH used to titrate positive electrode extract).

Voltage window	Negative electrode (mmol/kg)		Positive electrode (mmol/kg)	
	Pure N_2_	CO_2_/N_2_	Pure N_2_	CO_2_/N_2_
Not charged	6.83 ± 0.22			
0.5V	111 ± 16	189 ± 16	6.67 ± 0.00	6.67 ± 0.00
0.8V	159 ± 23	306 ± 28	15.6 ± 3.1	11.1 ± 3.10
1.0V	203 ± 90	452 ± 72	282 ± 18	179 ± 36
1.4V	133 ± 47	578 ± 31	268 ± 15	134 ± 13
1.0 V (discharged)	—	262 ± 34	—	9.50 ± 3.37

On the other hand, the pH of the extract from the positive electrode was only highly acidic (2.6 and 2.5) at 1.4 and 1 V. At 0.5 V and 0.8 V, significantly higher values of 6.5 and 5.1 were measured, respectively. At 0.5 and 0.8 V, the titration curves in N_2_ and CO_2_/N_2_ are nearly identical, while at 1.4 V the titrant consumption in N_2_ is considerably higher than in CO_2_ similar to 1.0 V. In all cases the equivalence point is near pH 7, showing the absence of H_2_CO_3_^*^ and HCO_3_^−^ as weak acids. The significantly lower titrant consumption at smaller voltages argues that the degassing at the positive electrode has a more pronounced effect at lower voltages (0.5 and 0.8 V).

## 3 Conclusion

Charging an SSA cell causes pronounced pH changes near the electrodes; as the electrolyte at the negative electrode becomes alkaline, and the positive electrode becomes acidic. The findings are supportive of the ionic liquid‐solid mechanism which assumes that the CO_2_ capture is driven by selective proton‐adsorption to the negative electrode. The high selectivity is due to the ability of protons to covalently bind to the carbon electrode upon negative charging. The removal of the protons from the chemical equilibrium leads to the dissolution and hydration of additional CO_2,_ generating bicarbonate and carbonate species near the negative electrode. These anions remain in the electrolyte near the negative electrode because there is no strong electrostatic driving force for these ions to migrate to the positive electrode. This is explained by the high concentration of the electrolyte (MgCl_2_), which leads to a potential drop primarily within the double‐layer, but not significantly across the separator. This idea is further supported by our recent study which showed that the CO_2_ sorption capacity increases with increasing electrolyte concentration [16].

The findings further explain why CO_2_ is desorbed from the positive electrode upon charging [12]. Due to the decreased pH near the positive electrode, the solubility of CO_2_ is decreased upon charging, and gaseous CO_2_ is released. The pH decrease has an onset between 0.5 and 0.8 V, possibly due to a proton‐releasing pseudocapacitive oxidation process occurring within that voltage range, and/or beginning water oxidation. Because of the insignificant amounts of (bi)‐carbonate present near the positive electrode, the adsorption of carbonate and bicarbonate to the double‐layer of the positive electrode is not plausible. The Cl^−^ions outnumber the bi‐(carbonate) ions by orders of magnitude, and there is no plausible driving force for a preferred adsorption of (bi)‐carbonate over chloride.

The findings from experiments at different voltage windows are in line with previously reported results that larger voltage windows led to higher CO_2_ sorption capacities. The results from this work can also explain why there is an enhanced sorption capacity when an SSA cell is cycled between −1 and +1 V. Upon a voltage change between −1 to + 1 V, the pH swings become larger, and additional CO_2_ is cycled. Finally, the results explain why the use of zinc instead of activated carbon as positive lead to an enhanced CO_2_ sorption capacity [32]. The use of zinc avoids the formation of acid near the positive electrode, and thus the desorption of CO_2_.

The results indicate the SSA has elements of pH swing carbon capture, and that the ionic liquid‐solid mechanism plays an important role. However, this does not rule out that the molecular liquid‐solid mechanism and the molecular gas‐solid mechanism also contribute. The difference to other pH‐swing carbon capture methods is that the acid and base are not generated through redox‐reactions, but capacitive charging and discharging. It should be noted here that pseudocapacitance could also contribute to the generation of acid and base at the electrodes. For example, the reduction of a carboxylic acid functionality to an aldehyde on the carbon surface is accompanied by proton acceptance Equation (13):

(13) $$-COOH_{\left(s\right)}+2e^{-}+2H_{\left(aq\right)}^{+}\to -CHO_{\left(s\right)}+H_{2}O_{\left(l\right)}$$

This process not only consumes protons from the aqueous electrolyte but also eliminates the acidic proton of the carboxylic acid group. Vice versa, aldehyde groups may be oxidized to carboxylic acids at the negative electrode, producing acid. Other pseudocapacitive effects may come from alcohol, keto, and alkene functionalities. Also, some oxidation of H_2_O to H_2_O_2_ at the positive electrode may occur, releasing protons. Hydrogen underdeposition at the negative electrode may contribute to hydroxide generation. The identification of the exact chemical changes on the electrode surfaces could be monitored IR‐spectroscopically in situ, and research into this direction is currently underway in our laboratory.

## Conflicts of Interest

Landskron has IP on SSA, is part of an SSA start‐up. The other authors declare no conflict of interest.

## Supporting information




**Supporting File**: smll73437‐sup‐0001‐SuppMat.docx.

## Data Availability

The data that support the findings of this study are openly available in Science DB at https://www.scidb.cn/en/s/NVzUJr.
