# Supplementary material for: Mechanistic Insights Into Supercapacitive Swing Adsorption via Acid–Base Titrations
Source: Small. 2026 Apr 20;22(32):e73437. doi: 10.1002/smll.73437 (PMC13244400; doi:10.1002/smll.73437)
Supplement: Supplementary file 1 — Supporting File: smll73437‐sup‐0001‐SuppMat.docx. [file SMLL-22-e73437-s001.docx]

**Supporting Information**

**Mechanistic Insights into Supercapacitive Swing Adsorption via Acid-Base Titrations**

Fareed Ul-Haq Khan & Kai Landskron^*^

Department of Chemistry, Lehigh University, 6 East Packer Avenue, Bethlehem, PA 18015

*kal205@lehigh.edu

**Experimental Methods**

**Electrode Preparation and Cell Setup**

Activated carbon electrodes were prepared from BPL carbon (Calgon Carbon Corp.) following our previously reported procedure^[1]^. Briefly, the carbon powder was mixed with conductive carbon black (VXCMAX22, Cabot Corporation), gluten (food-grade, Hodgson Mill) and PTFE (60% dispersion in water, Sigma Aldrich). 0.415 g of PTFE was dispersed in 30 ml of ethanol and sonicated for 10 minutes to ensure homogeneous dispersion, followed by addition of 4 g BPL carbon, 0.5 g gluten, 0.25 g carbon black and 20 ml ethanol. The solution was then heated for 2 hours at 65 ºC with constant stirring on a hot plate. After that, the solution was heated at 80 ºC while stirring to obtain a paste. The paste was then passed through a pasta machine to obtain flat sheets of ~0.7mm thickness which were cut into 2 cm x 2 cm size electrodes and dried in a vacuum oven at 100°C and 25 mmHg for 12 hours.

A pair of these carbon electrodes were assembled in a cell with a 3 cm x 3 cm (Whatman Grade 2, GE Healthcare Life Sciences) filter paper between them as the separator, and 3 M MgCl_2_ was used as the electrolyte. The bottom electrode was soaked in the electrolyte for 2 hours before assembly. A 4 cm^2^ carbon cloth (AvCarb 1071 HCB) was used as a gas diffusion layer above the top electrode. The cell assembly was surrounded by an EPDM rubber gasket (9.5 cm× 9.5 cm outer area, 8 cm × 8 cm inside area, 0.15 cm thickness, Fuel cell store) and sandwiched between titanium grade 7 plates (13 cm × 13 cm, 0.95 cm thickness). The cell was equipped with a central gas inlet port (1 mm diameter) located just above the top electrode that allows radial flow through the gas diffusion layer in the module. The effluent gas was collected at the exit port located at the corner of the top Ti plate, and passed through a drying tube, before entering a CO_2_ analyzer (Quantek Instruments, model 902P).

**Charging Procedure and CO₂ Adsorption**

The assembled cell was connected to a Gamry Potentiostat 3000 and subjected to cyclic charging and discharging. The cell was continuously fed with a CO_2_/N_2_ gas mixture (15% CO_2_ and 85% N₂ by volume, representative of a flue-gas concentration) at a constant flow rate of 1 sccm during the electrochemical cycles. The system was first cycled for ten full charge–discharge cycles to reach a steady state. A voltage window of 1.0 V (from 0 V up to –1.0 V) was used unless otherwise noted. Initially, the gas mixture was flown through the cell without any bias for 4 hours to allow for conventional adsorption of CO_2_ to the electrodes until the gas analyzer showed a constant value of 15% CO_2_. The cell was then charged to -1V at a rate of 50 mAg^-1^ and held at -1V for 30 minutes to allow complete saturation of the electrode with CO_2_. The CO_2_ concentration of the effluent gas was measured by the CO_2_ analyzer. After that, the cell was discharged to 0V at 50 mAg^-1^ followed by a 30-minute holding step for complete desorption of CO_2_ from the electrode. After these preconditioning cycles, the working electrode was charged once more to -1.0 V (a half-cycle, ending in the charged state) and held at that voltage for 30 minutes to saturate the electrodes with CO₂. The cell was then quickly disassembled to harvest the charged electrodes for analysis. For comparison, a control experiment was performed under identical conditions except using pure N₂ (0% CO_2_) as the feed gas; this control was used to probe the effects on the pH of in absence of CO_2_. As a further control, the pH of an uncharged electrode, which was never exposed to any gas, was also measured.

The system was also tested at the end of the discharge cycle (full cycle) to see if the pH returns to the original value (in the uncharged state). Further experiments were performed at different voltage windows to understand why the adsorption capacity is higher at larger voltage windows ^[2]^. These experiments were performed at -0.5V, -0.8V and -1.4V in addition to the standard -1V window. All the other parameters were kept the same.

**Electrode extraction and titration of the extracts**

The electrode was immersed in 5 mL of deionized water and ultrasonicated for 20 minutes. This process extracts the electrolyte within the electrode into the water. After sonication, the electrode was removed, and the pH of the remaining solution (which we refer to as the electrode extract) was immediately measured using a calibrated pH meter (Apera Instruments PH700 bench-top pH meter). **Figure S1** depicts the major steps performed in the experiments.


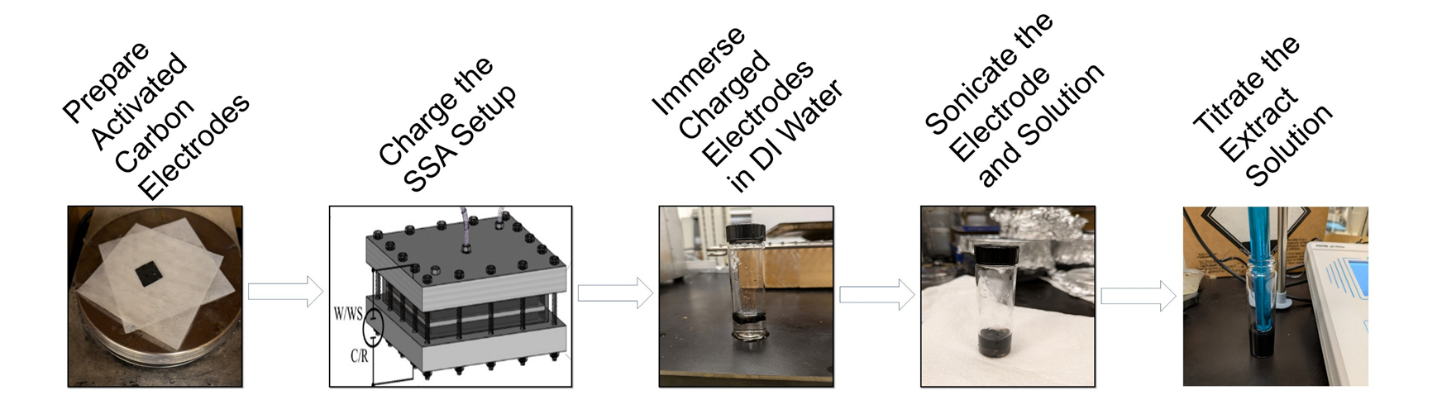


Figure S1: Steps involved in the pH titration for SSA mechanism studies

The pH changes in the extracts are due to the acid/base in the electrolyte inside the electrodes. To quantify their amounts, the extracts were titrated. For the solution from the negative electrode, which was found to be alkaline, a standard acid titrant 0.01 M HCl (Grainger Industrial) was added incrementally until the equivalence point was reached. Conversely, for the solution from the positive electrode, which was acidic, a basic titrant 0.01 M KOH (Grainger Industrial) was used for titration. Throughout the titrations, the pH meter was used to monitor the pH. From the volume of titrant added until the equivalence point, the molar amounts of acid or base in the solution were calculated. These are the molar amounts of acid and base that were initially present in the electrodes. For reference, we also performed the same extraction and titration on an uncharged electrode not subjected to any voltage or CO₂ exposure, termed the “never charged” electrode to measure the acidity or basicity arising from the carbon material’s inherent surface functional groups. This electrode was soaked in the electrolyte (3M MgCL_2_) for 2 hours and then placed in the DI water for the titration process. Plot for the titration of this electrode can be seen in **Figure S2**.

**
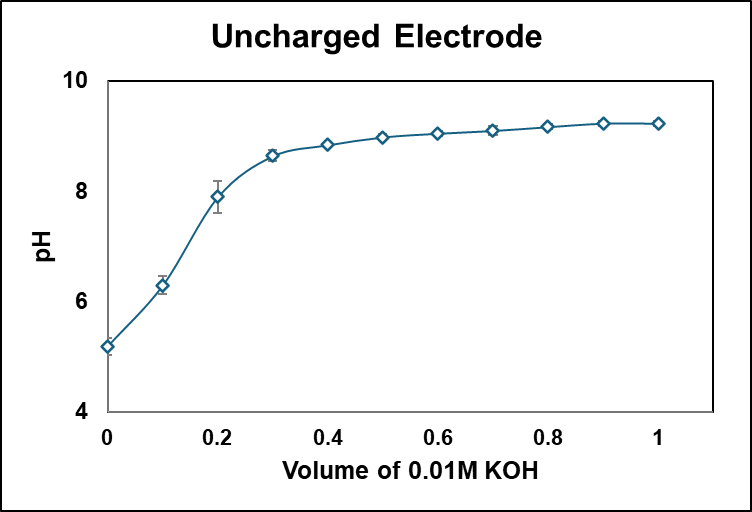
**

Figure S2: pH titration curve for the uncharged electrode. The error bars represent the average and uncertainty from three trials.

**Electrochemical Results**


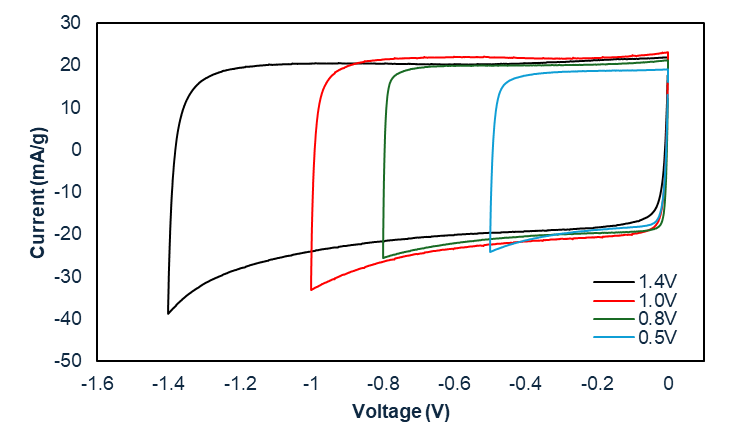


Figure S3: Cyclic Voltammograms at 1mV/s for different voltage windows


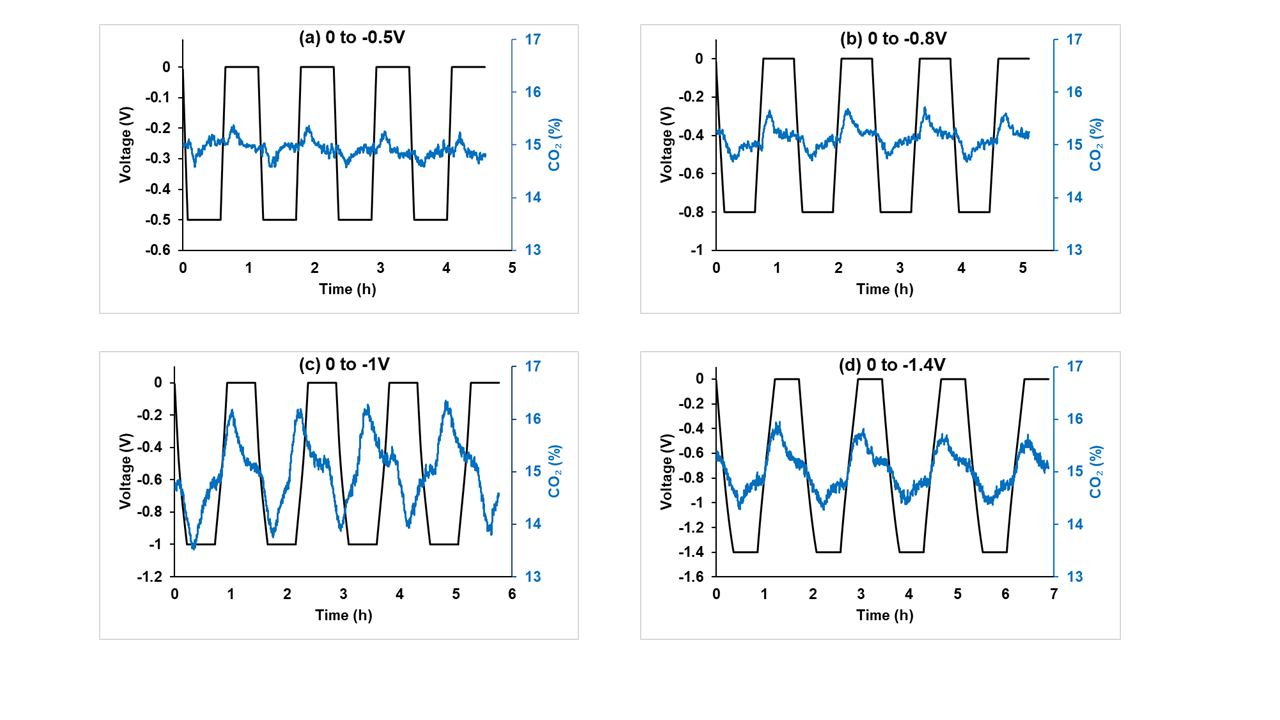


Figure S4: Voltage response (black) and CO2 concentration profiles (blue) at different voltage windows (a-d). 85%CO_2_/ 15%N_2_ mixture was used as feed gas at a flow rate of 1sccm for all experiments

***SSA Energetic Metrics***

The SSA energetic metrics^[3]^ include the calculation of equivalent series resistance (R_ES_, Ω cm^2^), gravimetric capacitance (C_s_, F g^−1^), charging time (t_g_, s), total charge stored during charging (Q_c_, C), total charge stored during discharging (Q_d_, C), coulombic efficiency (η_c_, %), energy efficiency (η_e_, %), and energy loss (ΔE, J). These metrics can be obtained from galvanostatic charge–discharge curves.

$$R_{ES}=\frac{V_{pstat}-V_{drop}}{I}$$

$$C_{s}=\frac{4.I.t_{g}}{m.\Delta V}$$

$$Q_{c}=\int_{0}^{t} Idt$$

$$\eta_{c}=\frac{Q_{d}}{Q_{c}}\times100$$

$$\eta_{e}=\frac{E_{d}}{E_{c}}\times100$$

$$\Delta E=E_{c}-E_{d}$$

Where *V*_pstat_ is the potentiostatic voltage (either 0 V or higher voltage (-0.5 to -1.4) V), *V*_drop_ is the voltage at the start of the charging or discharging step, *I* is the constant current, *t*_g_ is the charging time, *m* is the total mass of two electrodes, Δ*V* is the charging potential window, *E*_d_ is the energy delivered during discharging, and *E*_c_ is the energy consumed during charging. The capacitance calculations assume equal capacitance of the two electrodes.

***SSA Adsorptive Metrics***

The SSA adsorptive metrics^[3]^ include the calculation of the number of moles of CO_2_ adsorbed during the charging plus the holding step (n_a_, µmol), adsorption capacity with respect to mass (AC, mmol kg^−1^) of the gas-exposed electrode, adsorption rate (AR, µmol kg^−1^ s^−1^) and energy consumption (EC, KJ mol^−1^).

$$n_{a}=\frac{P}{RT}(\int_{0}^{t} f_{i}dt--\int_{0}^{t} f_{e}dt)$$

$AC=\frac{n_{CO2ads.}}{m_{top}}$

$$AR=\frac{n_{CO2ads.}}{m_{top}\times t}$$

$$EC=\frac{\Delta E}{n_{a}}$$

Where P is the pressure (1 atm), T is the temperature (296 K), R is the general gas constant, *f_i_* and *f_e_* are the influent and effluent gas flow rates, *m_top_* is the mass of top electrode, and t is the total charging time (including the holding step).

Table S1: Electrochemical results comparison at different voltage windows

| **Voltage Windows** | | **0.5V** | **0.8V** | **1.0V** | **1.4V** |
| --- | --- | --- | --- | --- | --- |
| **Energetic Metrics** | | | | | |
| R_ES_ (Ω.cm^2^) | at low V | 5.74 | 4.86 | 4.30 | 5.77 |
|  | at high V | 5.80 | 5.26 | 4.56 | 5.79 |
| Cs (Fg^-1^) | | 74.8 | 74.9 | 86.9 | 106 |
| t_g_ (s) | | 2058 | 2244 | 2490 | 2798 |
| ɳ_c_(%) | | 98.5 | 98.1 | 98.8 | 85.4 |
| ɳ_e_(%) | | 54.4 | 69.4 | 74.7 | 68.3 |
| ΔE (J) | | 0.41 | 0.72 | 1.20 | 2.77 |
| **Adsorptive Metrics** | | | | | |
| n_a_ (μmol) | | 2.24 | 3.79 | 9.66 | 16.5 |
| AC (mmol kg-1) | | 19.2 | 32.4 | 82.6 | 151.2 |
| AR (μmol kg-1 s-1) | | 9.33 | 14.0 | 25.8 | 54.0 |
| EC (kJ mol-1) | | 183 | 190 | 124 | 168 |

References

[1] C. Liu, K. Landskron, “Design, construction, and testing of a supercapacitive swing adsorption module for CO2 separation” *Chemical Communications* **2017**, *53*, 3661–3664.

[2] M. Bilal, J. Li, H. Guo, K. Landskron, “High-Voltage Supercapacitive Swing Adsorption of Carbon Dioxide” *Small* **2023**, *19*, DOI 10.1002/smll.202207834.

[3] F. Khan, M. Bilal, J. Li, X. Xu, K. Landskron, “Supercapacitive swing adsorption of CO_2_: advances and future prospects” *Trends Chem* **2025**, *7*, 43–55.
